# Therapeutics of Chronic Otitis in the Dog

**Published:** 1897-10

**Authors:** 


### ABSTRACTS FROM FOREIGN JOURNALS.

### GERMAN.

UNDER THE DIRECTION OF J. PRESTON HOSKINS,
PRINCETON, N. J.

THERAPEUTICS OF CHRONIC OTITIS IN THE DOG.—In the treatment of this disease many practitioners have doubtless applied a great number of remedies without attaining the desired result; and how unpleasant it is, after a time, to feel compelled to announce to the owner that the recovery of the dog is beyond hope. In the treatment of the disease, the causes and duration of the same, as well as the progress which it has already made, may well be taken into consideration; yet to establish the last point is not always easy, if not exactly impossible. While in the one case treatment with powder is given the preference, in the other the application of medicine in fluid form has proved itself more effective; and I take the liberty to describe the procedure, borrowed from the sphere of human medicine, with which a complete cure was quickly obtained in some very advanced cases. Yet, I should remark beforehand that my experiments were not numerous enough, from lack of material, to admit of a positive result, and I publish them now, so that a trial of the treatment may be made elsewhere, although in the few cases I thus treated the result was surprising.

The sick animal should be laid upon a table and held firmly by attendants. A muzzle is likewise absolutely necessary to prevent biting. The ear affected must then be thoroughly syringed out with lukewarm water, to remove entirely all accumulations formed by the existing inflammation. The greater the changes which have taken place, the longer must the syringe be used. The ear should then be thoroughly dried out by means of the pincers and a small piece of gauze, and, according to the size of the dog, from ten to twenty drops of a 3 per cent. solution of chromic acid be dropped into the ear-canal with the dropper. The fluid which has been thus dropped in is to be distributed well through the canal by kneading gently at the base of the ear for about two minutes; then follow up with repeated syringing with lukewarm water, and then close up the ear with a wad of cotton. This treatment should be repeated in two days, and I have obtained the most beautiful results in from eight to fourteen days after the treatment had been repeated three or four times, according to the extent and acuteness of the disease. After this treatment the animals usually shake the head violently, but only for a short time; and it often happens that the cotton wad falls out, yet this is of no great significance. On the first application of this remedy the diseased places become tinged with yellow from the chromic acid, and the extent of the inflammation can thus be readily determined. The application of this remedy is to be recommended only in chronic cases, for my friend and colleague, Wille, tried it in an acute case, and the trouble was augmented rather than decreased. It is also to be noted that the solution of chromic acid should be prepared fresh for every application, as it loses its effectiveness when kept standing.—*Wochenschrift* (Albrecht and Goring), in *Thierärztlches Centralblatt*, November 1, 1896.

